# Turning “Cold” Into “Hot” Tumors—Opportunities and Challenges for Radio-Immunotherapy Against Primary and Metastatic Brain Cancers

**DOI:** 10.3389/fonc.2019.00163

**Published:** 2019-03-19

**Authors:** Lisa Sevenich

**Affiliations:** Georg-Speyer-Haus, Institute for Tumor Biology and Experimental Therapy, Frankfurt, Germany

**Keywords:** GBM, brain metastasis, tumor immunology, immune therapy, immune checkpoint blockade, ionizing radiation, radiotherapy

## Abstract

The development of immunotherapies has revolutionized intervention strategies for a variety of primary cancers. Despite this promising progress, treatment options for primary brain cancer and brain metastasis remain limited and still largely depend on surgical resection, radio- and/or chemotherapy. The paucity in the successful development of immunotherapies for brain cancers can in part be attributed to the traditional view of the brain as an immunologically privileged site. The presence of the blood-brain barrier and the absence of lymphatic drainage were believed to restrict the entry of blood-borne immune and inflammatory cells into the central nervous system (CNS), leading to an exclusion of the brain from systemic immune surveillance. However, recent insight from pre-clinical and clinical studies on the immune landscape of brain cancers challenged this dogma. Recruitment of blood-borne immune cells into the CNS provides unprecedented opportunities for the development of tumor microenvironment (TME)-targeted or immunotherapies against primary and metastatic cancers. Moreover, it is increasingly recognized that in addition to genotoxic effects, ionizing radiation represents a critical modulator of tumor-associated inflammation and synergizes with immunotherapies in adjuvant settings. This review summarizes current knowledge on the cellular and molecular identity of tumor-associated immune cells in primary and metastatic brain cancers and discusses underlying mechanisms by which ionizing radiation modulates the immune response. Detailed mechanistic insight into the effects of radiation on the unique immune landscape of brain cancers is essential for the development of multimodality intervention strategies in which immune-modulatory effects of radiotherapy are exploited to sensitize brain cancers to immunotherapies by converting immunologically “cold” into “hot” environments.

## Introduction

Primary and metastatic brain tumors represent a challenging clinical issue. Glioblastoma (GBM) with an incidence of 2–3 per 100,000 population, is the most common primary brain tumor making up 54% of all gliomas and 16% of all primary brain tumors (1). Brain metastases (BrM), that most frequently arise from melanoma, breast- or lung cancers, are the most common intracranial tumor in adults and exceed the number of primary brain tumors by ~5-fold (2, 3). With the advent of improved control of systemic disease and increased life expectancy of cancer patients, the number of patients with brain metastases is rising (4). The development of cerebral tumors is associated with deteriorated quality of life due to headaches, epileptic seizures, and gradual cognitive impairment (5). Surgical resection, chemo- and radiotherapy (RT) remain the standard of care treatment for patients with brain tumors. Despite recent advances in the development of novel therapies against extracranial tumors, only very little progress has been made in the treatment of cerebral cancers. The majority of clinical trials with immunotherapies for GBM or BrM showed only moderate responses and did not significantly improve progression free survival (PFS) and overall survival (OS) (6). The lack of progress in the development of novel therapies for brain tumors can at least in part be attributed to the unique physiology of the central nervous system (CNS) and in consequence the highly complex brain tumor microenvironment (7). Brain tumors establish an immune suppressive tumor microenvironment that is characterized by high myeloid cell content together with relatively low tumor infiltrating lymphocyte (TIL) numbers and signs of T cell exhaustion (7). While immunotherapy alone fails to provide significant survival benefits for brain cancer patients, there is accumulating evidence, that adjuvant radiotherapy increases tumor immunogenicity and sensitizes brain tumors toward immunotherapy (8, 9).

The primary goal of radiotherapy is the induction of DNA damage in rapidly dividing tumor cells to induce different forms of cell death such as apoptosis or mitotic catastrophe (10, 11). In contrast to malignantly transformed tumor cells with impaired DNA repair mechanism, non-transformed stromal cells experience less damage given their post-mitotic state and intact DNA repair machinery (10). Although a link between irradiation and the immune system was proposed already 100 years ago (12), anti-tumor effects of radiotherapy were attributed to genotoxic effects on tumor cells, while effects on bystander cells were largely neglected for decades. Radiation dose and fractionation was therefore chosen to induce maximal damage in tumor cells and to spare bystander cells. However, traditional dose regimens might blunt important immune reactions directed against tumors. The discovery of immunogenic cell death (ICD) and abscopal effects provide formal proofs for immunological effects of radiation (13, 14). Abscopal effects describe the phenomenon that radiotherapy exerts anti-tumor effects in lesions outside the radiation field by triggering systemic anti-tumor effects (15). Therefore, exploiting the immune modulatory functions of radiotherapy represents an attractive tool to convert immunologically “cold” environments into “hot” environments to increase response rates of immunotherapy. This review will discuss preclinical and clinical evidence that support the applicability of radiotherapy as a sensitizer of immunologically inert tumors, such as GBM and BrM toward immunotherapy with a focus on immune checkpoint blockade (ICB). The field of radio-immunology is just at the beginning to understand the complex cellular and molecular effects of ionizing radiation (IR) on tumor cells and tumor-associated stromal cells that lead to more pronounced and long-lasting immune responses. In addition to clinical observations, it is important to employ preclinical models for systematic evaluation of different treatment regimens in terms of scheduling and dosage to maximize the synergy of radio-immunotherapy. Insight into cellular and molecular effects of radio-immunotherapy is critical to provide a strong scientific rationale for the development of multimodality intervention strategies.

Detailed understanding of the immune landscape of the central nervous system (CNS) at steady state and under pathological conditions is critical to appreciate immunological effects of radiotherapy in brain tumors. This review will therefore first summarize current knowledge on immune surveillance in the CNS and discuss how the development of primary and secondary brain tumors modulates the cellular composition of the TME and alters effector functions of tumor-associated immune cells. Based on this knowledge, different immunological aspects of brain tumors will be discussed to provide insight into the molecular basis of immunotherapy and radiotherapy in combination settings with a particular focus on differences in immune modulation depending on dose and fractionation of IR.

## Immune Surveillance in the Central Nervous System

**Figure 1:**
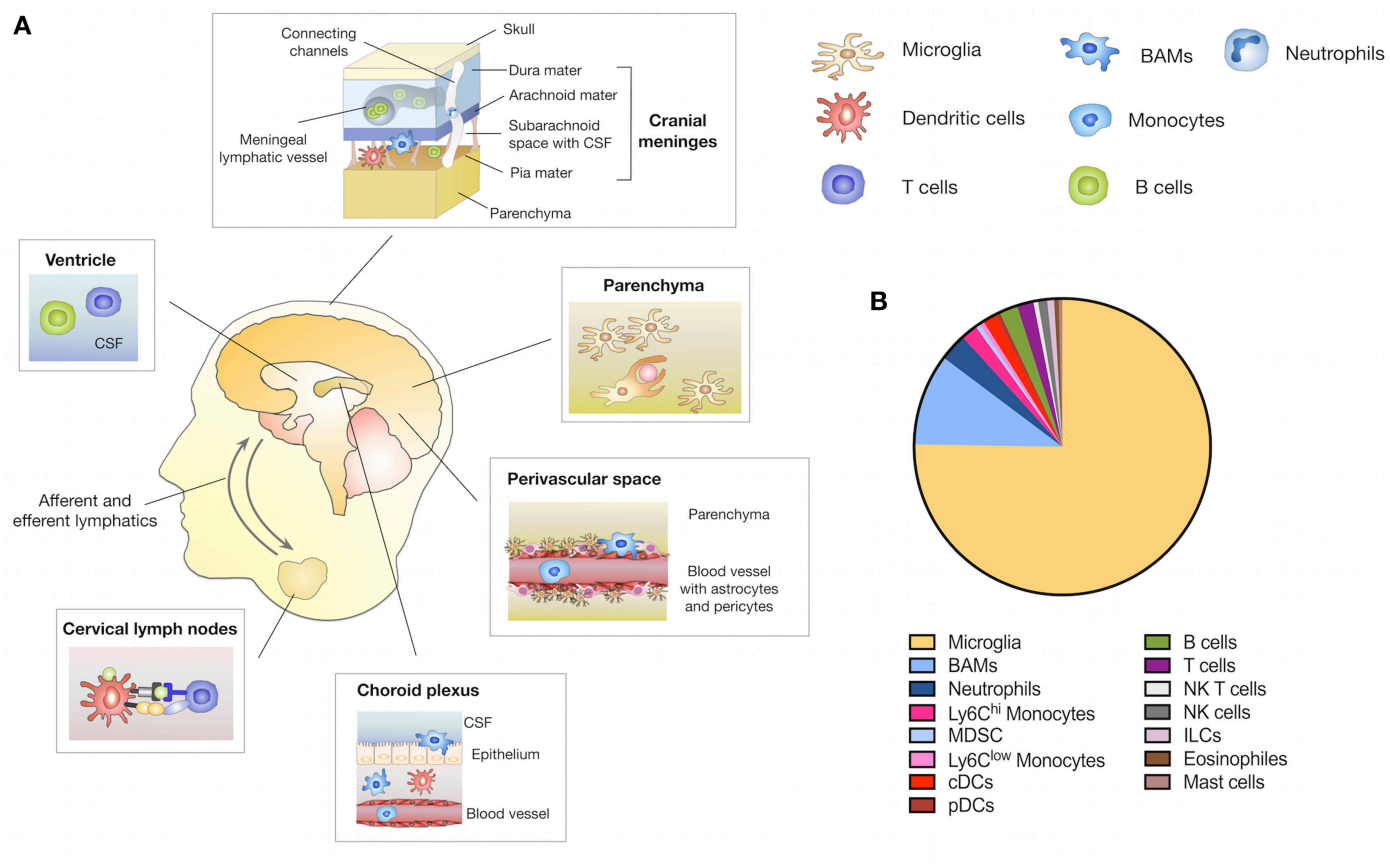
Immune compartments in the CNS. **(A)** The brain parenchyma is tightly shielded from the systemic immune system by the BBB and BCB that restricts the entry of peripheral immune and inflammatory cells. Yolk sac-derived microglia represent in the innate immune cell type within the brain parenchyma and exert key functions in host defense, immune surveillance and tissue homeostasis. In addition to the brain parenchyma, the CNS comprises structural interfaces that connect the CNS with the periphery, such as the choroid plexus, the meninges and the perivascular spaces. Border-associated macrophages (BAMs) are located in each of these niches. Circulating immune cells such as dendritic cells (DC), B- and T-cells are found in the CSF predominately in the meninges, ventricles and choroid plexus. A meningeal lymphatic system allows DC trafficking from the CNS to CNS draining lymph nodes i.e., cervical lymph nodes. Direct channels that connect the skull bone marrow with the dura enable neutrophils to migrate to the CNS as immediate responders to CNS damage. **(B)** Relative amount of distinct CNS immune cell populations located in the parenchyma and border areas of the CNS. Depicted amounts are based on data published in Mrdjen et al. (20).

The CNS has traditionally been regarded as an immune privileged site that is excluded from systemic immune surveillance (16). Several observations constituted the concept of the immune privileged status of the CNS. First it was noted that the CNS fails to elicit an immune response against immunogenic material that was implanted into the brain parenchyma when avoiding the ventricles and meninges (17, 18). Moreover, the presence of the blood-brain-barrier (BBB) or blood-cerebrospinal fluid barrier (BCB) as well as the absence of lymphatic vessels as a route to the lymph node for antigen presenting cells (APC) further underpinned the concept of the CNS immune privilege (16). However, more detailed insights into the anatomical structures of the brain that represent an interface between the CNS and the periphery led to recent revisiting of the immune privilege of the CNS (16, 19). The use of single cell sequencing and single cell cytometric approaches helped to elucidate the complexity of immune populations in the steady state CNS (20). In this regard it is important to discriminate between brain regions that are excluded from systemic immune surveillance such as the parenchyma and areas at the border between the CNS and the periphery, including the meninges and the choroid plexus. The presence of the BBB and BCB restrict the entry of immune cell-types as well as the exchange of macromolecules into the brain parenchyma under physiological conditions (16, 21). Host defense is therefore performed by microglia, the brain-resident macrophages that constitute the largest population of immune cells in the CNS (22, 23) (Figures 1A,B). Parenchymal microglia are long living myeloid cells with self-renewal capacity that arise exclusively from the yolk sac and populate the brain during embryogenesis before the establishment of the BBB (24–27). As the innate immune cell of the brain, microglia exert key functions in immune surveillance, resolution of infection, wound repair, phagocytosis and debris removal (28). Moreover, microglia are involved in maintaining tissue homeostasis by mediating synaptic pruning, myelo- and neurogenesis as well as neuronal apoptosis (29, 30). However, compared to other cells of the macrophage lineage, microglia show lower antigen-presenting capacity (20, 31). While the brain parenchyma is tightly shielded from the systemic immune system, there are routes that peripheral leukocytes can utilize to enter the cerebral spinal fluid (CSF), choroid plexus, meninges, and the perivascular space (32). Border-associated myeloid cells (BAMs) including meningeal macrophages (mMF), choroid plexus macrophages (cMF), and perivascular macrophages (pvMF) populate those specialized locations in the CNS (33) (Figures 1A,B). While microglia exclusively originate from yolk sac-derived progenitors, BAM progenitors are of mixed ontological origin deriving from the yolk sac during primitive hematopoiesis and the fetal liver or bone marrow during definitive hematopoiesis (33). Single cell sequencing and mass cytometry (CYTOF) approaches indicate that BAMs show distinct gene expression signatures compared to microglia (20). Importantly, certain subsets of BAMs showed high CD38 and MHCII expression indicating a role in antigen presentation (20). In addition to the tissue-resident macrophages, Ly6Chi and Ly6Clow monocytes as well as dendritic cells constitute the myeloid compartment of the CNS (20, 28). Monocytes and DC are primarily localized in the meninges and choroid plexus (34–36). It was recently demonstrated that myeloid cells, i.e., neutrophils migrate through vascular channels in the skull-dura interface, indicating a direct local interaction between the brain and the skull bone marrow through the meninges as a route for immediate response to brain damage (37).

Moreover, a range of lymphoid cells including B- and T cells as well as innate lymphoid cells (ILC), natural killer (NK), and natural killer T (NKT) cells constitute the lymphoid compartment in the CNS (20). Lymphocytes are absent from the brain parenchyma but can be found within the CSF of the meninges, choroid plexus and the ventricles. Importantly, the recent discovery that the CNS is directly connected to secondary cervical lymph nodes via a standard lymphatic drainage system fundamentally changed the concept of peripheral immune responses within the CNS (38, 39). There are three known routes by which intracranial antigens can traffic to CNS draining lymph nodes (40). The first is via ventricular and subarachnoid CSF that is able to cross the cribriform plate and enter the lymphatics draining into the deep cervical lymph nodes (41). Secondly, CSF is able to enter meningeal lymphatics located in the dura that also drain to the deep cervical lymph nodes (39). The third route results from parenchymal interstitial fluid trafficking through the basement membrane of the wall of capillaries and arteries of the brain (42). The first two routes are accessible to immune cells such as T cells, monocytes and DC as well as soluble antigens, while the third route is limited to soluble antigens (40).

The immune landscape in the CNS under steady state has recently been shown to be more complex than previously noted. Based on recent observations it was proposed to refer to the brain as an immunologically distinct rather than privileged site. Despite the description of the cellular constituents of the immune landscape in the CNS, it will be critical to evaluate to which extent the presence of lymphoid and myeloid cells in border-associated areas affects the immune privileged state of the brain parenchyma. In this regard, it is important to identify pathological stimuli that trigger infiltration of immune cells from border-associated areas as a route for immediate response or lead to recruitment of immune cells from the periphery and induction of a systemic response. There is accumulating evidence that different pathological conditions, including neurodegenerative disorders as well as cerebral cancers, induce fundamental changes in the cellular composition of the immune infiltrate and activation state of key players in neuro-inflammation (43). Importantly, recent studies revealed that in particular cells that are recruited from the periphery are implicated in the generation of an immune suppressive and cancer permissive environment, while brain-resident cells rather maintain host defense functions (31). This review will focus on tumor-associated inflammation in primary and metastatic brain cancers and highlight similarities and unique characteristics of immune responses that are provoked in the CNS during tumor progression. Understanding the complex immune landscape of brain cancers is critical to develop strategies to overcome the generation of an immune-suppressive environment and perturb traits of tumor cells to escape immune surveillance.

## Brain Tumors Establish an Immune-Suppressive Environment

**Figure 2:**
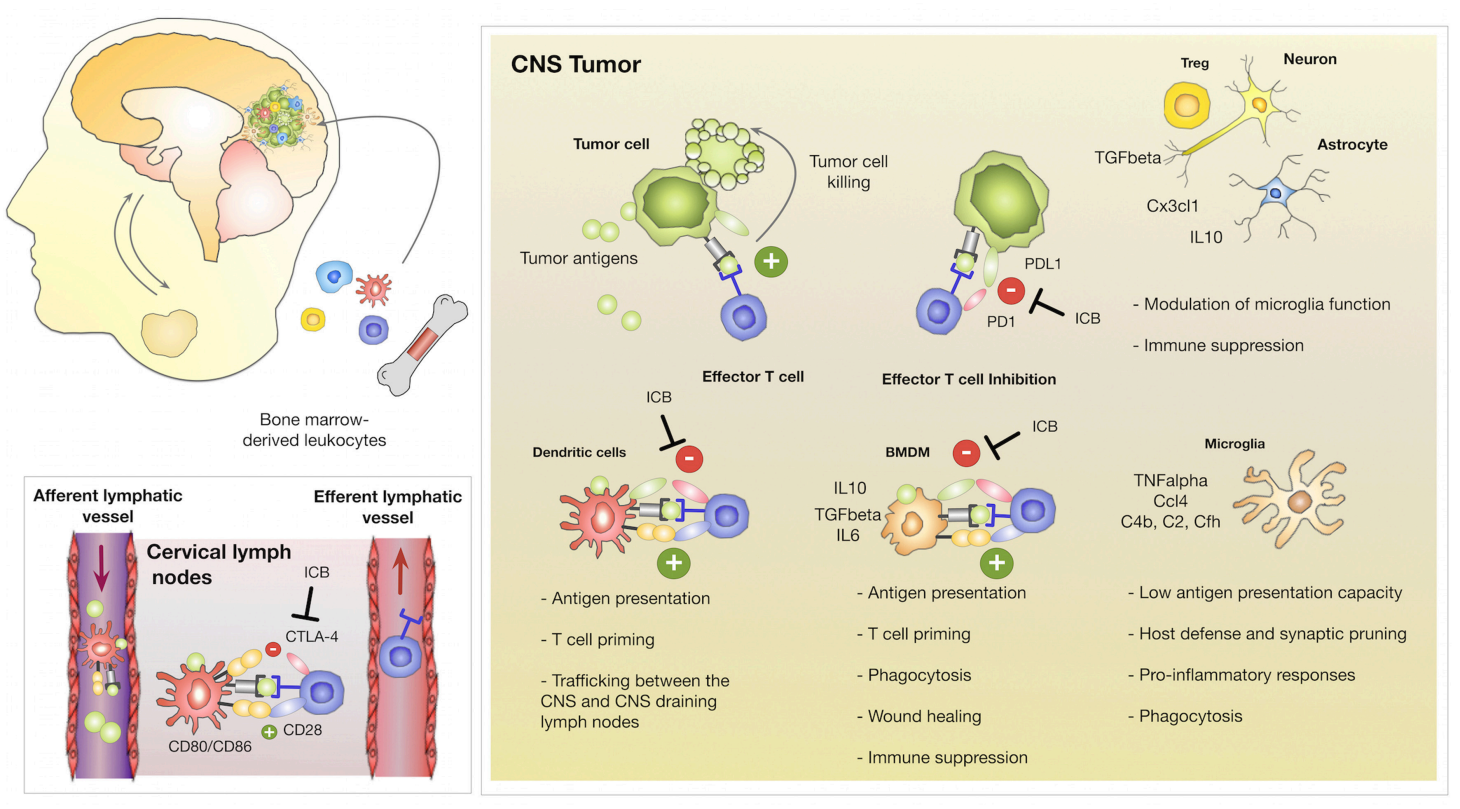
Innate and adoptive immune responses in CNS tumors. Brain tumors induce the recruitment of myeloid and lymphoid cells. Brain-resident and recruited cell types exert different functions within tumor-associated inflammation. Brain resident microglia maintain functions associated to their role as the innate immune cell of the CNS including host defense and synaptic pruning while bone marrow derived macrophages are associated with antigen-presentation, immune suppression and wound healing/tumor promoting functions. TAM-BMDMs express high levels of checkpoint molecules including PD-L1 to inactivate T cells. Dendritic cells traffic between CNS tumors and the cervical lymph nodes to prime T cells against tumor neo-antigens. T cells receive activating signals through interactions of the T cell receptor with antigens presented on MHC molecules and co-stimulation through interactions with CD28 and CD80/CD86. DC express the checkpoint molecule CTLA-4 that binds to CD28 on T cells to prevent activation of auto-reactive T cells. It remains unclear to which extent DCs activate or inhibit cytotoxic T cell functions in CNS tumors. Cytotoxic T cell responses are further inhibited by aberrant expression of checkpoint molecules on tumor cells as well as the secretion of immune-suppressive cytokines by Tregs, astrocytes and neurons. Checkpoint inhibitors (Immune Checkpoint Blockade; ICB) that block CTLA-4, PD-1, or PD-L1 unleash T cell effector functions to induce cytotoxic activity against tumor cells.

The development of primary and metastatic brain tumors disrupts the BBB leading to pronounced influx of blood-borne myeloid and lymphoid cells that are usually absent from the brain parenchyma. Tumor-associated macrophages (TAM) represent the most abundant stromal cell type in GBM and BrM often constituting up to 30% of the tumor mass (44, 45). Microglia and BMDMs share many phenotypic and functional similarities. The discrimination of both cell types in the context of brain cancers was previously challenging due to their overlapping marker expression and similar morphology in brain tumors. Lineage tracing approaches (46) and the recent discovery of specific markers (31, 47, 48) significantly contributed to our understanding of cell type specific functions of microglia and BMDMs during disease progression. It was long believed that tumor-associated microglia (TAM-MG) and tumor-associated bone marrow-derived macrophages (TAM-BMDM) exert similar functions in brain tumors. However, gene expression analysis of GBM-associated microglia and macrophages revealed that TAM-MG maintain gene signatures that are associated with house-keeping functions such as synaptic pruning and host defense and induce pro-inflammatory responses. In contrast, recruited TAM-BMDM showed gene signatures that are associated with wound healing, antigen presentation and immune suppression (31, 49) (Figure 2). Hence, functional differences of TAMs based on their ontological origin affect their contribution to disease progression and the ratio of TAM-BMDM to TAM-MG is expected to determine prognosis and therapeutic response especially of intervention strategies that aim to reinstate an effective anti-tumor immune response. In addition to evidence from the mouse models, single cell RNAseq analysis confirmed functional differences between TAM-BMDM and TAM-MG based on their ontological origin in human GBM (50). Interestingly, Müller et al. found that TAM-BMDM signatures correlate with significantly shorter survival in low-grade glioma (LGG) with similar trends in GBM, while there is no correlation between survival and TAM-MG signatures (50). Similar to GBM, it was also reported, that TAM-BMDM infiltrate BrM, although to a lesser extent (31). However, it remains unclear if BrM induce similar gene signatures in TAM-MG and TAM-BMDM as described in GBM. In addition to TAM-BMDM, tumor-infiltrating dendritic cells (DC) represent the most important antigen presenting cell type in brain tumors (51). The ability of DC to collect antigens in peripheral organs and to migrate to draining lymph nodes to activate and prime T cells is fundamental for cytotoxic T cell responses directed against specific antigens (52, 53). Given the immune privileged status of the brain, it remained unclear whether tumor-specific antigens in the CNS are surveyed by the immune system involving trafficking of DCs from CNS tumors to draining lymph nodes as well as trafficking of primed T cells from cervical lymph nodes into CNS tumors. To address the question on T cell trafficking, Prins et al., performed cell-tracking experiments to follow tumor antigen specific T cells after adoptive transfer of *in vitro* activated Pmel T cells (54). Imaging of T cell trafficking in this experimental system that is based on systemic vaccination revealed an early accumulation of T cells in all lymphoid organs including the cervical lymph nodes that drain the CNS and a subsequent accumulation in the bone marrow and brain tumors (54). Moreover, Garzon-Muvdi et al. employed an OVA-expressing GBM model with adaptive transfer of OT-1 T cells to identify the site of antigen presentation of tumor-antigens (55). The authors found proliferating OT1 T cells in cervical lymph nodes indicating that antigen presentation and T cell priming against tumor antigens might take place in the CNS draining lymph nodes (Figure 2). T cell expansion in the lymph nodes and anti-tumor effects were most pronounced in response to DC activation by the Toll-like Receptor (TLR)-3 agonist poly(I:C) in combination with PD-1 mediated immunotherapy (55), indicating that a strong proliferative stimulus is needed for effective T cell expansion. Moreover, it remains to be elucidated whether endogenous tumor antigens can elicit T cell priming or if this only occurs in response to highly immunogenic epitopes such as OVA. While the studies by Prins et al. and Garzon-Muvdi et al. provide evidence that under experimental conditions the proposed route of DC migration, T cell priming and local expansion might take place, it is important to acknowledge that the systems are based on strong experimental stimuli that are not expected in naturally grown CNS tumors. Hence, the formal proof of the route in which DCs migrate to cervical lymph nodes to prime T cells that subsequently traffic to CNS tumors to exert cytotoxic functions is still missing to date. While the question on the natural route for DCs and T cells remains to be addressed in CNS tumors, there is strong evidence that activated, cytotoxic T cells that infiltrate CNS tumors encounter a highly immune-suppressive milieu (56–59). Immune-suppression is particularly well-documented in GBM that are almost completely devoid of T cells (60). Quantitative deficits in the T cell compartment i.e., lymphopenia have been described for GBM patients since the late 1970 (61). It was recently demonstrated that glioblastoma as well as other intracranial tumors induce lymphopenia through sequestration of T cells in the bone marrow leading to a decline in T cell numbers at the tumor site and in lymphoid organs (62). Moreover, T cell apoptosis is induced through interactions of tumor cells via CD70-CD27 signaling (63, 64) or through astrocytes-derived FasL (65) at the tumor site. In addition to quantitative effects on T cells, qualitative deficits of T cells are a common phenomenon in patients with intracranial tumors (66). T cell dysfunction in brain tumors can be induced by a variety of mechanisms (67). High levels of immune-suppressive cytokines such as IL6, IL10, and TGFβ dampen T cell proliferation and effector functions (68). Tumor infiltrating lymphocytes (TILs) show high levels of PD-1, CTLA-4, LAG3, TIM3, TIGIT, and CD39 indicating T cell exhaustion (69–73). Regulatory T cells (Treg) comprise up to 30% of the TILs in GBM that further suppress T cell responses (57, 74). Tumor-associated macrophages and microglia have also been shown to support immune suppression and inhibit the expansion of CD4+ and CD8+ T cell expansion while inducing Treg production (31, 49, 75, 76).

The immune landscape of BrM and its consequences on systemic and CNS immunity are less well-characterized compared to primary brain cancers. The question, if immune responses in BrM are predominately driven by the tissue environment or if the cellular identity of the tumor of origin (e.g., melanoma, lung, renal or breast cancer) shapes the mode of inflammation, is currently a field of active research. In contrast to GBM, BrM show moderate or even pronounced T cell infiltration. Several studies reported that the extent and pattern of T cell infiltration depends on the primary tumor entity that metastasizes to the brain (77, 78). T cell density is highest in melanoma with a diffuse pattern throughout the metastatic lesion, while renal-, lung- and breast cancer lead to a moderate T cell influx and T cells are dominantly localized within the stromal compartments of the tumor (77). Data from different studies indicate that T cell exhaustion also appears in brain metastasis. For example, expression of PD1 has been found in ~63% of TILs in melanoma brain metastasis (79, 80). A study by Harter et al. demonstrated that high TIL level, PD1+/CD8+ and PDL1 staining were associated with smaller lesions, however there was no significant association with survival (77). In contrast, a study by Berghoff et al., reported a significant correlation of the density of CD3+, CD8+, and CD45RO+ TILs with favorable median survival (78). As observed in GBM, immune suppressive cell types such as Tregs have also been shown to infiltrate experimental models of metastatic melanoma, breast and colon cancer within the brain and were found in patient brain metastases (57, 81–84).

Taken together, thorough investigation of the tumor microenvironment in GBM and BrM indicate that brain cancers contain the cellular and molecular constituents for therapeutic intervention by checkpoint inhibition. However, clinical data revealed that checkpoint inhibitors as mono-therapy often fail to significantly improve survival rates (85, 86). A possible explanation for the inability of checkpoint inhibitors to reinstate an anti-tumor response might be that the majority of infiltrating T cells are bystander cells that are not directed against specific tumor antigens (87). However, it is also possible that T cells with anti-tumor activity are present within brain tumors, but local immune suppression efficiently blunts their cytotoxic activity even in the presence of checkpoint inhibitors (Figure 2). If this is the case, therapeutic strategies that block immune suppression are required to sensitize brain tumors toward immunotherapy. In this context, immunological effects of radiotherapy recently attracted attention and a series of clinical trials have been initiated to test the efficacy of radiotherapy in combination with immunotherapy. The next paragraph will summarize the current status of standard of care and discuss insights from clinical trials with a focus on trials with ICB in GBM and BrM.

## Clinical Management of GBM and BrM

### Standard of Care

The current standard of care for newly diagnosed GBM is maximal surgical resection with concurrent radiotherapy and temozolomide (TMZ) chemotherapy followed by 6 months of adjuvant TMZ treatment (88). However, despite multimodality therapeutic intervention, GBM has an almost 100% relapse rate with a median time to recurrence of 7 months (89). The clinical situation for patients with recurrent GBM is extremely dire. Surgery is only considered for ~25% of the patients and re-irradiation is only possible as a palliative option in rare cases (90). Moreover, response rates to chemotherapy including TMZ rarely exceed 10% and no effects on OS have been reported (91–93).

Previous radiation regimen for brain metastasis patients involved whole brain radiotherapy (WBRT) with fractionated doses of 30 Gy in 10 fractions or 20 Gy in 5 fractions. Different clinical trials report a cerebral response following WBRT in 60% of patients and tumor volume reduction after WBRT has been associated with better neurocognitive function and prolonged survival (94). Median survival following WBRT alone in patients with multiple brain metastasis ranges from 3 to 6 months, with 10–15% of patients still alive at 1 year. However, numerous detrimental effects of WBRT in terms of acute and delayed neurotoxicity such as leuko-encephalopathy and loss of memory function as well as radiation necrosis have been described (95, 96). Given the lack of survival benefit and both short- and long-term toxicities associated with WBRT, recent guidelines from the European Association of Neuro-Oncology (EANO) recommend a deferment and replacement of WBRT by stereotactic radiosurgery (SRS) for the treatment of patients with a limited number of brain metastases and/or favorable prognostic factors (97). SRS is a single high dose radiation treatment with high accuracy in placing the irradiation field on tumor lesions and improved protection of surrounding tissue. The treatment efficacy of SRS is similar to surgical resection of brain metastases, with local control rates ranging from 80–85% (98). Clinical data also show, that the use of SRS after surgical resection significantly lowers local recurrence compared to surgery alone and that it is associated with a decreased risk of cognitive decline compared to WBRT (99, 100). Controversy remains over potential differences of SRS plus WBRT compared to SRS or WBRT alone. A recent meta-analysis that compared the outcome of patients with one or more brain metastases revealed no differences on survival for patients with multiple metastases, while a WBRT plus SRS improved survival in patients with single metastasis. Moreover, WBRT plus SRS resulted in significantly better local tumor control than WBRT alone (101).

Given the dismal prognosis for GBM and BrM patients, in particular patients with recurrent GBM or patients with multiple BrM it is evident that improved intervention strategies are urgently needed to provide better care for brain cancer patients. The introduction of immunotherapy into the clinics for select cancer types led to new hope for an improved management of primary and metastatic brain cancers.

### Immunotherapy

Immune checkpoints are an important component of immune responses to keep cytotoxic activity of T cells under control to prevent autoimmunity. Cancers exploit this safety mechanism by up-regulation of checkpoint components on their cell surface to block T cell activity or by co-opting cells of the tumor microenvironment to establish an immune suppressive environment by dampening T cell responses. Checkpoint inhibitors unleash T cells from their inactive or exhausted state to induce anti-tumor responses (102) (Figure 2). To date, the most prominent examples have been antibodies that block the inhibitory immune checkpoint proteins cytotoxic T lymphocyte antigen 4 (CTLA-4), and PD-1 that are expressed predominantly on T cells, or PD-L1 that is expressed on different immune cells as well as aberrantly on tumor cells (103, 104). Consequently, successful immunotherapy by checkpoint inhibition relies on the natural ability of T cells to recognize and destroy malignant cells. While GBM and BrM are both characterized by highly immunosuppressive environments, GBM is further characterized by T cell exclusion and low mutational burden resulting in minimal neo-antigen generation (105). In contrast, BrM show moderate to high T cell content depending on the primary tumor entity and the majority of tumors that metastasize to the brain show high mutational load. However, it has to be taken into account that mutations that are found in brain metastasis are often not present in matched primary tumors (106). Data on brain metastasis patients are limited since those patients are often excluded from clinical trials. However, immunotherapies have demonstrated survival benefits for patients with tumors that frequently metastasize to the brain such as melanoma and NSCLC (107, 108). The use of checkpoint inhibitors in those patient cohorts allows for retrospective studies to evaluate the efficacy of checkpoint inhibitors against brain metastasis (109), which indicated efficacy of ICB in BrM. Given the potential beneficial effect of ICB in brain metastasis, a limited number of prospective trials have now been initiated to test the efficacy of immunotherapies in the treatment of brain metastases. First clinical trials to evaluate the efficacy of ipilimumab in patients with melanoma brain metastasis reported intracranial responses in 18% of patients with asymptomatic BrM without corticosteroid treatment while only 5% of symptomatic BrM patients on corticosteroid treatment showed intracranial responses (110). This finding further underpins the need that patients are not treated with corticosteroids at the time of ipilimumab treatment. Following clinical trials such as the ABC trial (NCT02374242) (111) and CheckMate-204 (112) aimed to test the efficacy of combining nivolumab and ipilimumab. Both trials report significant intracranial response rates of 46% in the combined treatment group compared to 20% in the nivolumab group in the ABC trial and 57% in the CheckMate-204 trial. Novel combinations are currently explored in clinical trials to further increase the intracranial response and to reduce adverse effects. For example, the activity and safety of the VEGF neutralizing antibody bevacizumab in combination with pembrolizumab or atezolizumab is tested in clinical trial for patients with untreated BrM (NCT02681549; melanoma and NSCLC and NCT03175432 BEAT-MBM; melanoma). Besides the effects of bevacizumab on angiogenesis, there is accumulating evidence, that VEGF blockade leads to reprogramming of the tumor microenvironment from an immunosuppressive to an immune permissive milieu, thus representing a promising combination together with immune checkpoint inhibitors (113). Results from these trials are still pending.

Although multiple factors indicate that GBM harbors intrinsic resistance against checkpoint inhibition as mono-therapy, pre-clinical testing showed promising results (114). The CheckMate143 trial (NCT0207717) was the first large-scale randomized clinical trial of PD pathway inhibition in GBM. However, treatment with the PD1 blocking antibody nivolumab failed to extent OS in patients with recurrent GBM, leading to a termination of this trial arm (86). In particular in the context of GBM it is important to take into account that in addition to the immunosuppressive tumor microenvironment, GBM patients often receive TMZ that is known to cause lymphopenia and permanently affect numbers of memory T cells (115). Moreover, corticosteroids such as dexamethasone are commonly used in the treatment of GBM patients to control cerebral edema. However, as already mentioned for BrM patients, corticosteroids are known to adversely affect the efficacy of immunotherapies (116).

In sum, the extent of immune suppression that is established in GBM and BrM together with effects from standard of care treatment that further dampens immune responses might ultimately prevent effective immunotherapies. Therefore, it is important to develop improved intervention strategies that overcome current obstacles to successful immunotherapy in cerebral tumors. In addition to the recently initiated clinical trials that aim to test the efficacy of the combination of different checkpoint inhibitors, there is increasing interest in the potential synergy of radiotherapy and immunotherapy.

### Radio-Immunotherapy

Data from retrospective trials suggest that combinations of immunotherapy and radiotherapy significantly increase response rates and show effects on overall survival. For example, Knisely et al. reported that melanoma patients that received ipilimumab plus WBRT achieved longer median survival compared to WBRT alone [21.3 vs. 4.9 months] and a greater 2-year survival rate [47.2 vs. 19.7%] (117). Similarly, ipilimumab plus SRS was shown to increase OS from 5.3 to 18.3 months, while in this study no survival benefits for the combination of ipilimumab plus WBRT was reported (118). Sharverdian et al. reported that within the patient cohort that was enrolled in the KEYNOTE-001 trial (NCT01295827), NSCLC patients that received radiotherapy before pembrolizumab showed better PFS and OS compared to patients who did not receive radiotherapy (119). Ahmed et al. recently reported data from melanoma BrM patients that received nivolumab plus SRS demonstrating high rates of local BrM control of 91% and 85% at the 6 and 12 months follow-up (120). A central question that remains to be addressed in combination trials is the timing of each component for optimal outcome. Possible regimens comprise concurrent, sequential or neo-adjuvant application of the treatment modules (121). So far, the results suggest that the optimal schedule is tumor type and immunotherapy dependent. However, to date, the majority of trials report data that provide evidence for a benefit of concurrent schedules (122) and lowest response rate if radiotherapy is given after the immunotherapy. For example, a study of patients with melanoma brain metastasis showed that concurrent immunotherapy with anti-PD-L1 and anti-CTLA-4 showed improved response rates if immunotherapy was given within a time frame of 4 weeks after radiation compared to treatments that were more than 4 weeks apart (122). Dovedi et al. demonstrated that acquired resistance to fractionated radiation could be overcome by PD-L1 blockade using syngeneic mouse models of melanoma, colorectal and triple-negative breast cancer. However, the effect was only apparent if treatments were applied either concomitantly with or at the end of radiation. The effect was lost if PD-L1 blockade was given 1 week after radiotherapy (123). In contrast, a retrospective analysis of data from 758 patients suggested an improvement of OS with concurrent ICB and RT and hypo-fractionated RT particularly when immune checkpoint inhibition is started at least 1 month before RT, implying a benefit to commence ICB prior to RT (124). To date, pre-clinical and clinical evidence that optimal scheduling of radio-immuno-therapy critically affects the therapeutic response largely stems from studies on extracranial tumors and must be carefully considered for individual cancer types and different checkpoint inhibitors. Defining the optimal schedule for primary and metastatic brain tumors will require carefully designed prospective clinical trials in combination with systematic preclinical testing or mathematical modeling approaches as recently proposed by Serre et al. (125). While there are several ongoing clinical trials that aim to compare the efficacy of immune checkpoint inhibitors in combination with either WBRT or SRS, there are only few trials that are specifically designed to evaluate how different schedules affect safety and efficacy of combined treatment. One phase II clinical trial at the University of Michigan Cancer Center (NCT02097732) that considers the timing of immunotherapy is evaluating the efficacy of an “induction” of ICB prior to SRS [2 doses of ipilimumab prior to SRS, 2 doses of ipilimumab after SRS] vs. “no induction” [SRS first, followed by 4 doses of ipilimumab (3 mg/kg)]. In the future, more clinical trials that address the question on optimal scheduling will be required for conclusive results.

**Figure 3:**
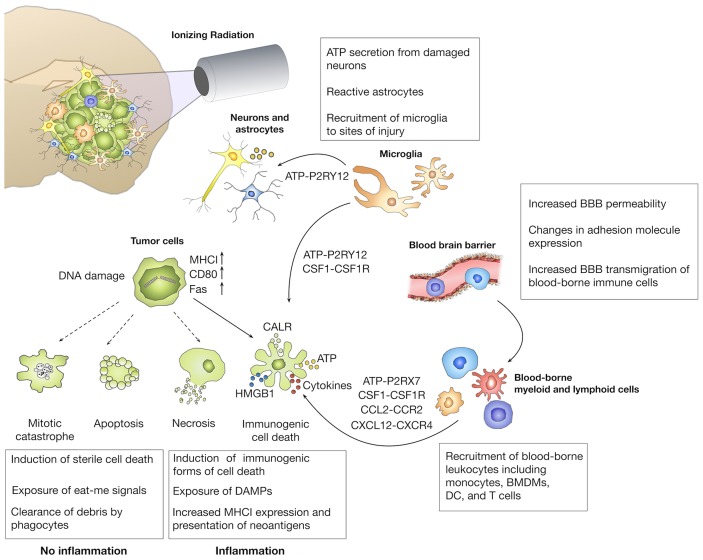
Immune modulatory effects of radiotherapy. The primary aim of radiotherapy is the induction of DNA damage in malignant cells. Depending on the extent of DNA damage, different forms of cell death are induced. The induction of immunogenic cell death (ICD) has been linked to the exposure of danger-associated molecular patterns (DAMP) that induce the recruitment and affect effector functions of brain-resident and recruited immune cells. Radiotherapy can also increase the immunogenicity of tumors by inducing presentation of neo-antigens by upregulation of MHC I molecules. In addition to effects on tumor cells, ionizing radiation also affects tumor-associated stromal cells including cells of the blood vessels and immune cells that further contribute to the establishment of radiation-induced immune responses.

In sum, data from clinical and pre-clinical studies indicate that radiotherapy can act as a sensitizer for immunotherapy. Conventional fractionation takes advantage of the higher radio-sensitivity of tumor cells compared to normal cells with respect to DNA repair and cell cycle regulation. However, whether conventional fractionation represents the optimal strategy to maximize synergy with immunotherapy remains unclear to date. Moreover, it will be critical to evaluate whether radiation dose and fractionation that is optimal to induce an immune response in the CNS, is tolerated by the sensitive brain tissue. In order to optimize radiation dose and fractionation as well as scheduling for therapeutic application, it is essential to gain detailed insight into the molecular basis of genotoxic and immune modulatory effects of radiotherapy. The following paragraph will summarize current knowledge on effects of radiotherapy that result from direct damage on tumor cells (i.e., different forms of cell death) with subsequent effects on the inflammatory response against tumors. Moreover, local and systemic immune responses can also be modulated by radiation-induced changes in different cell populations of the tumor microenvironment (Figure 3). Direct and indirect effects on tumor cells and tumor-associated immune cells together determine the extent by which radiotherapy increases immunogenicity of tumors and the synergy between radio- and immunotherapy.

## Molecular Basis of Immune Modulatory Effects of IR

### Radiation-Induced Immune Responses Depend on the Type of Cell Death by Which Tumor Cells Are Killed

The central dogma of traditional radiobiology states that effects of radiation on tumor cells are primarily due to the generation of double strand breaks that lead to the induction of different forms of cell death including apoptosis, necrosis, autophagy, or mitotic catastrophe (Figure 3). Apart from the notion that necrosis elicits inflammation due to the release of cellular content, radiotherapy has long been regarded as an immunologically inert process. While immunological effects of radiotherapy were neglected for decades, several discoveries established a link between the immune system and the ability of radiotherapy to achieve tumor control. Stone et al. demonstrated already end of the 70s, that the radiation dose that is required to control tumor growth was twice as high in immune-compromised mice compared to immune-competent mice (126). Moreover, the occurrence of abscopal effects in which tumor control is achieved in lesions outside the radiation field provides further proof for the contribution of the immune system in tumor control (127). The discovery of immunogenic cell death (ICD) as a molecularly defined processes that leads to priming and activation of immune cells recently led to a paradigm shift. ICD is characterized by the cell surface translocation of calreticulin (CRT), the extracellular release of HMGB1 (High motility group box 1) and of adenosine triphosphate (ATP) (128). Radiotherapy has been shown to induce all three arms of ICD and is therefore regarded as a potent inducer of ICD (129). Different doses or fractionations are believed to induce different forms of cell death (130), and thus modulate downstream cellular responses. Radiation regimens that induce immunologically silent forms of cell death, i.e., apoptotic cell death are therefore not expected to synergize with ICB, while doses and fractionation that trigger inflammatory responses could be used as immune-modulators to induce additive effects of radiotherapy and immunotherapy (131).

### Radiation Increases Recognition of Tumor Antigens by the Immune System

Within the process of immune evasion, tumor cells acquire traits that mask the tumor from immune surveillance and destruction. The immune escape stage is characterized by up-regulation of inhibitory ligands and cytokines, reduced MHCI expression, and increased number of suppressive cell types such as Tregs (132). Radiotherapy has been reported to unmask the tumor and thus make it visible again for the innate and adaptive immune system (133). Radiation can up-regulate MHCI expression on the tumor cell surface to enable better antigen presentation of tumor-specific peptides for recognition by cytotoxic T cells (134). Mutational load of tumors is known to correlate with therapeutic response rates to immunotherapy (135–138). Identification of mutations that drive anti-tumor responses would therefore be of great benefit to identify patients with a high chance of responding to immunotherapy. Moreover, radiation-induced DNA damage can cause an increase in mutational load (139). While an increase in mutational burden might enhance tumor aggressiveness, it might also generate neoantigens that can be recognized and targeted by the immune system (140). Indeed it was demonstrated that IR induces novel peptide synthesis in tumor cells and enhances antigen presentation by MHC class I molecules (134, 141). On the other hand, there is evidence that brain tumors show higher systemic tolerance than tumors at extracranial sites. For example, Jackson et al. employed the B16 melanoma model to compare cytotoxic responses against tumors in the CNS and in the periphery. The study showed that CNS melanomas were more tolerogenic than tumors in extracranial sites due to antigen-specific CD8 T cell depletion leading to impaired systemic antitumor immunity (142). The authors concluded, that the observed T cell dysfunction was mainly caused by elevated levels of microglia-derived TGF-β (142). Interestingly, it was demonstrated that the effect of systemic tolerance was reversible by radiotherapy and vaccination.

### The cGas-STING Axis in Anti-tumor Immunity

Radiation-induced DNA damage that causes leakage of DNA into the cytosol is known to be sensed by the stimulator of interferon gens (STING) leading to the activation of innate and adaptive immune responses (143). The STING pathway has originally been described as a host defense mechanism to protect organisms against infection with DNA pathogens. When cytosolic DNA is detected, the product of cyclic GMP-AMP synthase (cGAS), cyclic GMP-AMP (cGAMP) activates STING. STING induces the transcription of type I interferon genes via a cascade that involves the STING downstream factors Tank binding kinase (TBK), interferon regulatory factor 3 (IRF3) and nuclear factor kappa light chain enhancer of B cells (NFkB) (144). A growing body of literature suggests that the STING pathway plays a central role in anti-tumor immunity and its expression is lost in several cancer types including colorectal cancer and melanoma (145, 146). While several studies linked radiation-induced STING activity to type-I-interferon mediated anti-tumor immunity, there is also evidence that STING activation could drive immune-suppression and radio-resistance via CCR2 mediated recruitment of MDSCs (147). Given the complex cellular and molecular interactions of STING mediated immunological effects, clearly more systemic studies are needed to gain comprehensive mechanistic insight.

### Dose and Fractionation Are Critical Parameters for Effective Induction of Immunogenicity

Determining the optimal scheduling for radio-immunotherapy is a major challenge for the field and requires carefully designed prospective clinical studies together with comprehensive studies in animal models to test effects of different treatment regimens (148, 149). As discussed above, data from retrospective and prospective clinical trials suggests that treatment schedules in which radiotherapy was given as concurrent, sequential, or neoadjuvant therapy lead to different therapeutic efficacy. Several preclinical studies compared single high-dose with fractionated radiation for their ability to induce immune responses. For example, in a B16-OVA model, both single dose (15 Gy) and fractionated radiation (5x3 Gy) increased the generation of antigen-specific T cells. However, the single 15 Gy dose generated more tumor-infiltrating T cells than conventional fractionation (150). Later it was demonstrated, that the immune response triggered by ablative radiation doses was abrogated by conventional fractionation (151). Moreover, Camphausen et al. demonstrated in a model of Lewis lung carcinoma that 5 × 10 Gy induced more robust abscopal effects than 12 × 2 Gy (152). However, hypo-fractionation might not be favorable when combined with immunotherapy. Dewan et al. demonstrated in a breast cancer model that an abscopal effect was only induced in response to fractionated radiation, not single dose radiation when combined with CTLA-4 inhibition (153). A potential explanation for dose dependent effects of radiation was recently provided by the balance between activation of cGAS-STING signaling vs. Trex1 activation (154). Extremely high single doses (20-30Gy) were shown to blunt immunogenicity by the induction of the DNA exonuclease Trex1. Trex1-mediated degradation of cytosolic DNA consequently abrogates cGAS-STING activation and downstream IFN type1 production. In this study, CTLA-4 blockade did not synergize with high dose irradiation to induce abscopal effects. However, knockdown of Trex1 reinstated synergistic effects of anti-CTLA-4 in combination with high dose radiation (20Gy) (155).

### Radiation Modulates the Cellular Composition of the Tumor Microenvironment and Affects Effector Functions of Immune Cells

Another important factor that determines synergy of radio-immunotherapy is the cellular composition of the tumor microenvironment (156). As discussed in the paragraph above, in particular the tumor microenvironment of brain tumors represents a highly complex milieu with brain resident and recruited immune cells (7). Brain tumors are known to establish immune-suppressive environments that are characterized by high myeloid cell content and low percentage of CD8+ effector T cells. Several studies demonstrated that radiotherapy induces increased influx of immune cells into brain tumors. This effect can in part be attributed to effects on the vasculature (157, 158). IR also has profound effects on the secretion of cytokines that serve as chemo-attractants for different immune cells including DC and macrophages (159). In addition, IR has been shown to affect key effector functions such as phagocytosis, antigen presentation, and cytotoxicity and alters activation states of immune cells (160–162). Moreover, radio-sensitivity of T cells has to be taken into account when testing optimal dosage and fractionation. Since immunotherapies rely on functional T cells, their ablation or inactivation is expected to abrogate critical anti-tumor immune responses. Tumor-infiltrating T cells are exposed to radiation and it has been shown that conventional 2 Gy doses given once daily can inactivate T cells (163). This effect is also evidenced by the occurrence of lymphopenia as a common adverse effect associated with whole brain radiotherapy (164) that could significantly dampen anti-tumor immune responses.

Taken together, radiation dose and fractionation have profound effects on the induction of genotoxic and immunogenic effects. Systematic interrogation of the dose dependency of immune responses directed against different cancer types is needed to determine optimal regimens to increase the immunogenicity of tumors and boost the immune system for effective anti-tumor responses that synergize with immunotherapy.

## Combination of IR and ICB—Future Perspectives

To date, clinical and pre-clinical data suggest that combining radiotherapy with immunotherapy show higher efficacy compared to mono-therapies. These results represent promising first steps in the quest for improved treatment options for brain cancer patients. However, it is also evident that many hurdles exist that prevent higher response rates and more sustainable anti-tumor reactions. While individual patients show prominent cerebral responses, significant effects on overall survival are rarely reported. This indicates that the pressure of the CNS to establish an immunosuppressive environment is dominant over the attempt to unleash the immune system by immune checkpoint blockade. Based on our current mechanistic understanding of the cellular and molecular drivers of immune-suppression in the steady-state CNS and in the context of cerebral cancers, different approaches appear as viable strategies to overcome the highly immune-suppressive environment in the CNS. Based on the results that TAM-BMDM rather than TAM-MG are implicated in tumor-promotion and immune-suppression, selective depletion or blockade of TAM-BMDM recruitment could lead to more effective T cell activation and execution of anti-tumor effector functions. In addition, systems that would allow more efficient recruitment of T cells into CNS tumors could significantly boost cytotoxic T cell responses directed against tumor neo-antigens. A recent study by Samaha et al. reported the engineering of T cells with an Activated Leukocyte Cell Adhesion Molecule (ALCAM) homing system (HS) (165). In this approach, CD6 (the ligand for ALCAM) was re-engineered to trigger initial anchorage to ALCAM followed by adhesion to ICAM1 expressed on cancer endothelium. Cytotoxic HS T cells infiltrated brain tumors after intravenous injection and showed potent anti-tumor activity. Other strategies that aim to convert immune-suppressive milieus into inflamed environments might utilize neutralizing antibodies against suppressive cytokines such as TGFβ, IL10, or IL6. Moreover, activation of adenosine signaling has been associated with immune-suppression and acquisition of resistance against immunotherapy in different cancer types including melanoma (166–169). Pharmacological inhibition of enzymes that process ATP into adenosine, i.e., CD39/Entpd1 and CD73/Nt5e or targeting of adenosine receptors are currently evaluated for their potential to block the conversion of a purine-driven, pro-inflammatory environment into an adenosine-driven, immune-suppressive milieu (170–173). Another promising strategy could employ Trex1 inhibitors to prevent the degradation of cytosolic DNA to more efficiently induce cGAS-STING-IRF signaling to trigger innate immune responses (155). Overcoming the immune-suppressive environment appears to be one of the limiting factors for successful immunotherapy against brain cancers. However, it is also important to keep in mind, that immune suppression is an important safety mechanism that protects the brain from excessive inflammation. Inflammatory responses are often associated with swelling that would harm the delicate structures of the CNS and ultimately lead to brain damage. The increased risk of auto-immunity has been reported in several clinical trials with combination of different ICB. The most recent data on the CheckMate143 trial report that 9 of 10 patients treated with a combination of nivolumab and ipilimumab experienced grade 3 or 4 adverse events with 4 of 10 patients discontinuing therapy due to side effects (83). Therapeutic strategies that aim to convert immune suppressive milieus into inflamed environments should therefore be carefully considered and the potential risk of inducing autoimmunity should be evaluated. Detailed mechanistic insight into pathways that are implicated in cancer-associated immune-suppression and inherent or acquired resistance against brain tumors will hopefully lead to the development of novel multimodality intervention strategies that meet the safety and efficacy criteria for the induction of more efficient and long-lasting anti-tumor immune responses in GBM and BrM patients.

## Concluding Remarks

A close link between radiotherapy and the immune system has been proposed already 100 years ago by Russ and Murphy (12). Murphy's observation that “large doses of x-rays, by destroying the immune conditions, will favor the growth of tumors while small doses, by producing immune conditions will help to overcome the tumor” closely resembles the current view on effects of RT on cancer-associated inflammation (174). Yet, immunogenic effects of radiotherapy have been neglected for decades. The introduction of immunotherapy into the clinic and more detailed molecular insights into the underlying mechanism of immunogenic effects of radiation have recently attracted attention to radiotherapy as a potent modulator of cancer-associated inflammation. While immunotherapy is highly effective for patients with inflamed or hot tumor environments, large patient cohort remain unresponsive to immunotherapy due to intrinsic or acquired resistance. Although we are just at the beginning to understand the cellular and molecular basis that distinguish responders from non-responders, accumulating clinical and pre-clinical data indicate that immunogenic effects of radiotherapy convert cold into hot environments and thus sensitize unresponsive tumors toward immunotherapy. Our current knowledge on radio-immunotherapy against brain cancers is largely based on retrospective clinical trials or pre-clinical studies on animal models. The initiation of clinical trials and pre-clinical studies that aim to systematically evaluate the effects of different fractionation and treatment regimens is needed to provide deeper insight into optimal schedules to induce synergy between immunotherapy and radiotherapy. While there are certainly indications that favor specific treatment regimens, it is still too early to draw conclusions on effects on PFS or OS. To further improve the response rate, it will be critical to identify molecular pathways that determine the mode of immune responses and to develop strategies that efficiently induce anti-tumor immune responses without the risk of inducing auto-immunity.

## Author Contributions

The author confirms being the sole contributor of this work and has approved it for publication.

### Conflict of Interest Statement

The author declares that the research was conducted in the absence of any commercial or financial relationships that could be construed as a potential conflict of interest.
